# The unknown agents of European cooperation, and their future

**DOI:** 10.1007/s12027-021-00659-5

**Published:** 2021-03-29

**Authors:** Alex Brenninkmeijer

**Affiliations:** the European Court of Auditors, Luxembourg, Luxembourg

**Keywords:** European cooperation, Agencification, Network development, Governance, Accountability, Performance

## Abstract

Since the Maastricht Treaty, the EU has created 37 agencies and bodies covering nearly every aspect of our daily lives. However, agencies have always played a supporting role, serving national interests as well as those of Brussels, and their pivotal role in bringing those interests together is often overlooked. As agencies have increasingly been used as a solution for addressing the challenges facing Europe, their relationship with the European Commission, the Member States and EU citizens also needs to evolve as a model for European cooperation. A few guiding principles are relevant for this purpose, and have already been set out in a performance audit entitled “Future of EU agencies – Potential for more flexibility and cooperation” for which I had the pleasure to serve as the reporting Member to the European Parliament.

## The phenomenon of ‘agencification’ in EU public administration

In 2020, the European Court of Auditors published its first comprehensive performance audit on the development of EU agencies. Throughout the audit, much of the discussion with the European Commission was about the significant differences between agencies and the regulatory framework designed to characterise them as individual going concerns. Executive versus decentralised, self-financed versus EU-financed, regulatory versus non-regulatory, agencies versus bodies: the list goes on. One consistent feature, though, is the phenomenon of ‘agencification’ in EU public administration, which reflects the non-linear process of European integration – the shift of decision-making from national to supranational – involving an ‘intermediary agent’, and at the same time the growing need to tackle the shared global challenges facing all countries in what is a relatively small continent. In 2020, EU agencies outnumbered European Commission Directorates-General by 37 to 33. With a tiny fraction of EU resources (around 3%) and staff (around 13%), EU agencies have acquired influence over every aspect of our daily lives, and go about their work without much public attention.^1^

## More network development in a global context

When looking at the development of EU agencies today, it would be interesting to imagine the situation 30 years from now. The 2050 perspective on 2020 would recognise a timeline in which individual agencies had been created since the Maastricht Treaty to meet specific societal needs and where these agencies became cohesive units in a network due to their interdependent responsibilities in a more connected global environment. Such network development is not new, and can be compared with the network development of European universities in the 1990s or the European judicial system in the 1980s. In such a network, agencies are evolving into a more complex form of public administration, and could be viewed as *common centres of expertise and networking*.

*Common* refers to the different interests an EU agency is supposed to represent; *centre of expertise* means the need for an agency to advance knowledge and innovation, failing which it would become obsolete to its ‘creators’; and *networking* recognises that an agency increasingly has to cooperate with other agencies or partners at international level. The true value of EU agencies as *common centres of expertise and networking* has also manifested itself since the Brexit negotiations, when the focus was on how the UK could continue to be a committed partner by sharing expertise and networking despite its diverging interests; in other words, seeking to maintain influence through its presence in EU agencies. Over the years, EU agencies have built up IT infrastructure and other networking skills of immeasurable strategic importance; for sensible policymakers, such assets are too valuable not to be used to full advantage.

## The interplay between EU agencies and society

As EU agencies continue to evolve, the interplay between those agencies and society is increasingly important. One novel feature of EU agencies is the fact they were created at arm’s length from national and EU authorities, and so were granted sufficient creative scope to tailor their organisation and mandate to the political circumstances. However, whatever the impact of such creative scope on their daily operations, EU agencies – like any other public organisation – are answerable to citizens, and being an ‘intermediary agent’ still means doing the job that citizens expect national and EU authorities to do (even if they have not always lived up to expectations).

Due to their ‘mixed’ status, EU agencies face particular challenges in establishing a trust-based relationship directly with citizens. Although accountability is one aspect of trust, EU agencies have always reflected the positions of their partner Directorate-General in the European Commission which speaks for them at European Parliament or Council hearings. Although transparency is also an aspect of trust, a public record is rarely kept of the decisions taken by the EU agencies’ management boards. These boards, which sometimes have as many as 30 to 35 members, may cast votes, but the votes are not binding on the very institutions their members represent, i.e. the Member States, the European Commission and the European Parliament. This means that management board decisions cannot serve as a legal basis for enforcement. Lastly, although effectiveness is another aspect of trust, the media have long regarded the EU agencies as speaking on behalf of the European Commission, while their own successes and failures in achieving or influencing policy have escaped major criticism or analysis.

## The ownership of EU agencies

To whom, then, do EU agencies owe their performance as ‘intermediary agents’? The ‘creators’ of EU agencies have been arguing for their fair share of ownership by virtue of their intricate relations with the agencies. In practice, the partner Directorate-General in the European Commission (or ‘partner DG’, a term that is more neutral than ‘parent DG’) requires a hierarchical interaction with the EU agencies falling within its policy area. This interaction entails more formal control than pragmatic support, and covers issues that are crucial to the existence of EU agencies, such as resource allocation, task planning and reporting. In cases where an EU agency falls within the policy area of multiple partner DGs, uncoordinated communication and control from these DGs may hamper the agency’s performance. Furthermore, the European Parliament, when approving legislative proposals, often expects EU agencies to do more than they are legally obliged to. As information on policy achievements is scarce, the European Parliament struggles to send more cheques to the very areas where they are needed most. In addition, the Member States, through their representation on the agencies’ management boards and in the Council, can veto their own support for EU agencies. As EU agencies have little access to national politics, they need to rely on the national authorities’ willingness to cooperate, including those agencies which may been seen as competitors.

These intricate relations need new room to grow if EU agencies are expected to perform better, and, as their number has increased, the European Commission, the European Parliament and the Member States all seem to want to adopt a more hands-on approach. In 2012, the European Commission, the European Parliament and the Council of the EU issued a joint statement to strengthen oversight and to harmonise management and reporting. The question is, however, whether a one-size-fits-all framework is the future for EU agencies. For example, standardised templates are being produced with hundreds of performance indicators covering areas as diverse as gender balance and environmental footprints. While such detailed information across EU agencies may serve various management purposes, it does not answer the simple and yet fundamental question that citizens are most interested in: *how successful have the agencies been in achieving the purpose for which they were created*? This purpose is a matter for policy discussion, and can be justified by insight not only into individual agencies but also into their dynamics with other players in the same policy area. Recognising network development between EU agencies comes closer to a more dynamic clustering approach. Here, the network becomes the interface between the EU agencies and their ‘creators’ or ‘institutional partners’; horizontal integration and diversification between agencies is possible for each policy discussion; and instead of focusing on the specific performance indicators or activities of an individual agency in a ‘silo’, performance can be managed at macro level through the network.

## Network development as part of a lifecycle

Network development between EU agencies can also be viewed from the perspective of the agencies’ lifecycle. ‘Lifecycle’ means the creation, operation and possible closure of agencies over time. One theory, which, although sensitive, is frequently applied in the private sector, is the S-curve (or sigmoid growth curve): this illustrates the likely growth of a business, which starts out slowly, picks up speed during a period of rapid growth, and then tapers off as growth slows. Once a business reaches the last stage, investors usually switch to a new one. In the world of EU agencies, such an approach is politically and socially inconceivable. Over the years, none of the EU agencies have been closed, merged or significantly repurposed, except for the European Agency for Reconstruction, which was set up in 1999 and wound up in 2008. In the last decade, the European Commission twice proposed that agencies should be merged for the sake of synergy and efficiency, but failed to obtain European Parliament’s approval. The only soft option left seems to be to devise a new governance model for EU agencies that have developed rapidly in their complex environment and whose relations with institutional partners and citizens increasingly need to be reoriented in order to embark on a new phase of growth.

Interestingly, digitalisation offers many opportunities for a new governance model. More shared and real-time access to IT infrastructure, virtual means of communication, and big data may change how resources and tasks can be organised between EU agencies. Given their limited size and relatively recent origin (within the last two decades), many EU agencies can digitalise faster than their partner DGs or national counterparts. As an example, the European Court of Auditors is exploring a new relationship with EU agencies, based on automated and real-time financial audit processes facilitated by uniform IT systems in the executive agencies, and by using exogenous data other than official documents to corroborate findings in performance audits based on big, open data sources. While the Member States experience low absorption rate of EU funds and lack of project implementation, it might be worth considering the possibility of giving EU agencies a lead in investing in projects to improve digital infrastructure and governance, especially if they are organised in a bottom-up way that encourages participation by national authorities. Furthermore, the European Commission needs to use this new governance model to shift the focus from *data and information* to *knowledge and insights*, something that would enable it to concentrate on policy discussions about the purpose of EU agencies.

## A new governance model based on flexibility, cooperation and expertise

A few ideas are key to the new governance model, as implied in the earlier concept of *common centres of expertise and networking*, and the first of these is flexibility. Flexibility means not only resource flexibility (more or fewer resources), but also policy flexibility. EU agencies need to be able to accommodate policy changes and clarifications, and so their relevance and coherence needs to be evaluated regularly and in a targeted way. This is not currently the case. Many agencies do not have evaluation requirements or, if they do, evaluations are so rare, so general in terms of objectives, and so isolated within the remit of a single agency that they contribute little to policy discussions. Moreover, when new agencies are created, their impact on the mandate of existing agencies is not always properly addressed in the evaluations (or, in this case, the more technical term used by the European Commission is ‘impact assessment’). An illustration of this is a recent reservation expressed by the European Commission’s Regulatory Scrutiny Board (the advisory body to the College of Commissioners for the creation of a new agency) concerning the evaluation that paved the way for a European Labour Authority (ELA) in 2019. The reservation concerned the relationship between the ELA and the other four agencies falling within the same policy area of one partner DG. As a result of Covid-19, there is political momentum for an additional European Biomedical Advanced Research and Development Agency (BARDA). Here again, careful account should be taken of the impact on the mandate of existing health agencies, and the possible lessons learned from the network of agencies.

The second idea is cooperation. Cooperation means overcoming bureaucracy and focusing on simplification to achieve common goals. Less formal agency-to-agency contact – but more informal people-to-people contact – may enhance how relationships are built up between EU agencies and other institutional partners. Cooperation also means the ability to engage in conversation without apprehension. On many occasions, EU agencies can feel pressure – under the watch of their partner DG – not to cross the line of entering into binding agreements with international partners. By way of example, EU agencies are required to describe their strategy for international cooperation according to the European Commission’s guidelines, namely by ‘*highlighting that agencies remain within their mandate and the institutional framework, and that they do not appear as representing the European Union*’. In reality, cooperation has a much broader sense than signing binding texts. Very often, EU agencies may have to interact with international partners due to the nature of their work (e.g. the European Food Safety Authority, the European Medicines Agency, Europol and Eurojust, to name but a few of those that are more active).

Cooperation between EU agencies also needs to reach another level. So far, synergies have been sought primarily in horizontal administrative processes such as IT and HR. Policy coordination and cooperation is a more promising area, and EU agencies can share views and need to learn to find their voice. Even agencies that do not seem very policy-driven (such as the Translation Centre for the Bodies of the European Union, the European Training Foundation and the European Foundation for the Improvement of Living and Working Conditions) can actively contribute to cooperation on policies such as the Green Deal and Covid Recovery. Cooperation can also offset the imbalance between some EU agencies that operate in the same policy area but are exposed to different sides of the story, such as Frontex in the area of border control (‘surveillance’ and ‘guards’) and EASO in the area of asylum (‘victims’ and ‘rehabilitation’). In short, policy cooperation is crucial to EU agencies’ success, and needs to be fostered by their institutional partners. As far as EU citizens are concerned, individual EU agencies succeed only if the policy as a whole succeeds.

The third idea is expertise. Expertise means that EU agencies need to know their areas better. For example, the work of the European Aviation Safety Agency is regarded as an international standard. Expertise also means that EU agencies can mobilise intelligence easily within their network, including from national experts, and that they can reach out to data industries in order to build up capacity for foresight and for input into policy formulation. It is a common misconception that policy design is different from policy implementation, and that there is a clear division of responsibilities between the European Commission (for policy design) and EU agencies (for policy implementation). In practice, the two activities go hand in hand. Many implementation issues and the way achievements are measured need to be reflected in a policy’s design; at the same time, however, the way a policy is designed depends on the entity or entities that implement it. This means that EU agencies are also responsible for the success or failure of a policy.

## The future of EU agencies as common centres of expertise and networking

To conclude, EU agencies have long been the unknown agents of European cooperation. Many significant tasks have been delegated to them by national and EU authorities which maintain intricate relationships with the agencies. While these relationships are not always fruitful, EU agencies have learnt to become effective mediators: they find common ground for what is possible for the different interests they represent. As EU agencies continue to evolve, they also seek to reorient how they interact with citizens, with their institutional partners, as well as with each other. They have engaged in a type of network development that offers many opportunities for a new type of governance. It is important for such development to be recognised and analysed as an alternative model of public administration. While digitalisation can help to shape this new type of governance, some ideas – such as flexibility, cooperation and expertise – are key to its success, thus truly making EU agencies *common centres of expertise and networking*. The goal is therefore to build agencies that are more policy-driven, outward-looking, and future-oriented.

## Footnotes

1 EU agencies are active in aviation (EASA), health and food (ECHA, EMA, EFSA), vocational training (Cedefop, ETF), equal opportunities (EIGE), the financial and banking sectors (EBA, ESMA, SRB), the environment (EEA), animal and plant protection (EFCA, CPVO), and migration and border control (Frontex, EASA), to name but a few.

